# An IP3R3- and NPY-Expressing Microvillous Cell Mediates Tissue Homeostasis and Regeneration in the Mouse Olfactory Epithelium

**DOI:** 10.1371/journal.pone.0058668

**Published:** 2013-03-13

**Authors:** Cuihong Jia, Sebastien Hayoz, Chelsea R. Hutch, Tania R. Iqbal, Apryl E. Pooley, Colleen C. Hegg

**Affiliations:** 1 Department of Pharmacology and Toxicology, Michigan State University, East Lansing, Michigan, United States of America; 2 Neuroscience Program, Michigan State University, East Lansing, Michigan, United States of America; 3 Center for Integrative Toxicology, Michigan State University, East Lansing, Michigan, United States of America; National Institutes of Health, United States of America

## Abstract

Calcium-dependent release of neurotrophic factors plays an important role in the maintenance of neurons, yet the release mechanisms are understudied. The inositol triphosphate (IP3) receptor is a calcium release channel that has a physiological role in cell growth, development, sensory perception, neuronal signaling and secretion. In the olfactory system, the IP3 receptor subtype 3 (IP3R3) is expressed exclusively in a microvillous cell subtype that is the predominant cell expressing neurotrophic factor neuropeptide Y (NPY). We hypothesized that IP3R3-expressing microvillous cells secrete sufficient NPY needed for both the continual maintenance of the neuronal population and for neuroregeneration following injury. We addressed this question by assessing the release of NPY and the regenerative capabilities of wild type, IP3R3^+/−^, and IP3R3^−/−^ mice. Injury, simulated using extracellular ATP, induced IP3 receptor-mediated NPY release in wild-type mice. ATP-evoked NPY release was impaired in IP3R3^−/−^ mice, suggesting that IP3R3 contributes to NPY release following injury. Under normal physiological conditions, both IP3R3^−/−^ mice and explants from these mice had fewer progenitor cells that proliferate and differentiate into immature neurons. Although the number of mature neurons and the in vivo rate of proliferation were not altered, the proliferative response to the olfactotoxicant satratoxin G and olfactory bulb ablation injury was compromised in the olfactory epithelium of IP3R3^−/−^ mice. The reductions in both NPY release and number of progenitor cells in IP3R3^−/−^ mice point to a role of the IP3R3 in tissue homeostasis and neuroregeneration. Collectively, these data suggest that IP3R3 expressing microvillous cells are actively responsive to injury and promote recovery.

## Introduction

Adult progenitor cells reside in the basal compartment of olfactory epithelium (OE) and allow neurogenesis to occur throughout adult life. The two types of progenitor cells, horizontal and globose basal cells, consistently turnover and are multipotent, generating both neurons [1] and non-neuronal cells (sustentacular or microvillous cells) [2]. Basal cell proliferation and neuronal differentiation in the OE is tightly regulated by signals derived from a niche defined by the extracellular matrix of the basement membrane, growth factors released by surrounding cells, and the nearby vasculature [3]. The neuroproliferative peptide neuropeptide Y (NPY) is localized in IP3R3-expressing microvillous cells [4], [5] and to a smaller extent to sustentacular cells [6], [7]. These non-neuronal cells, components of the basal cell niche, have cell bodies located in the apical layer and cytoplasmic extensions that terminate in the basal cell layer. NPY stimulates proliferation of basal cells in vitro via a NPY Y1 receptor-activated extracellular signal-regulated kinase signaling cascade [6], [8], [9]. A significant reduction in basal cell proliferation occurs in NPY- [6] and NPY Y1 receptor-deficient mice [8]. Clearly, NPY promotes proliferation in the olfactory system. However, the autocrine or paracrine signaling pathways involved in NPY release in the olfactory system have not been elucidated.

IP3 is a second messenger that activates IP3 receptors (IP3Rs) that release intracellular calcium to regulate physiological processes including cell growth, development, sensory perception, neuronal signaling, and exocrine secretion [10]–[13]. Mammalian IP3Rs (types 1–3) are differentially expressed in the CNS, with type 1 and 3 IP3Rs expressed in neurons and type 2 IP3R expressed predominantly in glia [14]. In the OE, IP3R3s are expressed on a microvillous cell subtype [4], [15]. In general, IP3Rs are expressed on the endoplasmic reticulum, nucleus, plasma membrane, nerve terminals, and secretory chromaffin granules [16]. IP3- and IP3R3- mediated calcium signaling has been shown to have a role in secretion of secretory granules in endocrine and neuroendocrine cells [16]. Moreover, olfactory mucus secretion is decreased in the nasal glands of mice lacking type 2 and type 3 IP3 receptors [17], suggesting a secretory role for IP3Rs in the OE.

Given their expression of NPY [4], [5], we hypothesized that IP3R3-expressing microvillous cells secrete the NPY needed for both the continual maintenance of the neuronal population and for neuroregeneration following injury. We recently described the chemoresponsiveness of IP3R3-expressing microvillous cells in an IP3R3-knockout/tauGFP-knockin mouse [15]. Using this mouse model, we demonstrate that IP3R3s mediate NPY release and have an important role in the maintenance of progenitor cells and in injury-evoked adult neurogenesis.

## Materials and Methods

### Animals and Ethics Statement

Neonatal (postnatal day 1–5) and adult (6–8 weeks) male Swiss Webster and C57BL/6 mice were obtained from Charles River (Portage, MI). The IP3R3-tauGFP mouse (provided by Dr. Diego Restrepo, University of Colorado Denver, Aurora, CO), has the first exon of the Itpr3 gene replaced by the coding region for a fusion protein of tau and green fluorescent protein [15]. In the heterozygous IP3R3^+/−^ tauGFP^−/+^ mice (denoted IP3R3^+/−^), there is one normal IP3R3 gene and one IP3R3-tauGFP transgene, and in homozygous IP3R3^−/−^ tauGFP^+/+^ mice (denoted as IP3R3^−/−^), the biallelic expression of the IP3R3-tauGFP transgene eliminates IP3R3 expression. All efforts were made to minimize the number of animals used and their suffering. All procedures were conducted in accordance with the National Institutes of Health Guide for the Care and Use of Laboratory Animals as approved by Michigan State University Institutional Animal Care and Use Committee (#08/09-132-00).

### Measurement of NPY Release

OE slices (400 µm) were collected as described previously [7] from neonatal Swiss Webster, C57BL/6 and IP3R3^−/−^ mice (both sexes) only, as the bone has not yet calcified. For each treatment group 3–4 slices were used as 1 replication and experiments were repeated 3–8 times. When possible, each treatment group had OE slices obtained from a single neonate, although care was taken to match the size and level of the OE slices across treatment groups such that the amount of tissue incubated was approximately the same. Whole turbinates were collected from anesthetized (65–80 mg/kg ketamine +5–10 mg/kg xylazine) adult Swiss Webster mice. Each adult treatment group consisted of 1 turbinate and experiments were repeated 8 times. Slices or turbinates were placed on a cell culture plate insert (Millicell-CM PICMORG50, Milipore Corp., Billerica, MA) and incubated at 37°C with 5% CO_2_ in neurobasal media with 0.02 g/L B-27 supplement, 0.01 g/L penicillin/streptomycin and 0.01 g/L L-glutamine (Invitrogen, Carlsbad, CA) in the absence or presence of P2 purinergic receptor agonists (20, 250 and 500 µM ATP and 50 µM BzATP), P2Y receptor agonist (50 µM UTP) and P2X receptor agonist (50 µM αβ-MeATP). Some slices were pre-treated with vehicle (0.2% DMSO), PLC inhibitor (100 µM U73122) or IP3 receptor inhibitor (100 µM 2-APB) 15 or 45 min prior to agonist incubation. Conditioned media were collected 1 hr after agonist treatment. In some experiments, slices were incubated with caged iso-IP3/PM (1.5 µM caged IP3, Enzo Life Sciences, Plymouth Meeting, MA) or vehicle (0.09% pluronic acid +0.45% DMSO) for 45 min and illuminated with unfiltered light from a xenon arc lamp for 30 min to uncage IP3. The media were collected 1 hour later. Conditioned media were concentrated with a SVC200H Speed Vac Concentrator (Savant Industries Inc., Farmingdale, NY). The levels of NPY in conditioned media were measured by NPY ELISA kit (Peninsula Laboratories, San Carlos, CA) following manufacturer’s protocols. Data is reported as ng NPY/OE slice for neonates, or pg NPY/mg OE protein as measured using a BCA protein assay kit (Pierce Biotechnology, Rockford, IL) for adults.

### Western Blot and PCR Analysis of IP3R3-tauGFP Mouse OE

OE tissue from anesthetized (4% isoflurane) adult male C57BL/6 mice and both sexes of IP3R3^+/−^ and IP3R3^−/−^ mice was collected from each side of the septum and stored separately at −80°C. OE from one side of the septum was used for Western blot to measure IP3R3 protein levels and the other was used for PCR to identify the presence of the IP3R3 gene. Primers (Integrated DNA Technologies, Inc., Coralville, IA) for detection of IP3R3 transcripts were forward (5′- GGTGAGTGAGCCTAGGGCAAAGAGA -3′) and reverse (5′- GCCTGGAGGATGCTTGGAGAAGA -3′), and primers for GFP and CRE were as described previously [15]. For Western blot analysis, OE tissues were processed following the protocol described previously [18]. After incubation with blocking buffer (5% BSA), the membranes were probed with mouse anti-IP3R3 antibody (1∶1000, BD Biosciences, San Jose, CA) overnight at 4°C. The membranes were washed and then incubated with HRP-labeled secondary antibody (1∶2000, Jackson ImmunoResearch Laboratory, West Grove, PA). Immunoreactive proteins were detected with a chemiluminescence reagent (ECL, Amersham Biosciences, Piscataway, NJ) and then exposed to Kodak X-ray film. Membranes were probed a second time with mouse anti-actin antibody (1∶2000, Santa Cruz Biotechnology, Santa Cruz, CA).

### In vivo Drug Administration

Anesthetized (4% isoflurane) adult male C57BL/6 mice and both sexes of IP3R3^+/−^ IP3R3^−/−^ mice (n = 3–4 mice/group) aspired a bolus of ATP (400 nmol/kg), NPY (4 nmol/kg) or an equivalent volume (50 µl) of saline placed on the nares. Some mice intranasally aspired IP3 receptor inhibitor (2-APB, 400 nmol/kg) 30 min prior to ATP or saline. In the injury studies, saline vehicle or satratoxin G (100 µg/kg) were aspired followed by daily intranasal treatment with saline vehicle or ATP (400 nmol/kg) for 2 or 5 days. In order to detect BrdU-incorporation, some animals received two BrdU injections (i.p., total 144 mg/kg) at 6 and 3 hours prior to tissue collection.

### Immunohistochemistry

Frozen coronal OE tissue sections (20 µm) from adult male C57BL/6 mice and IP3R3^+/−^ and IP3R3^−/−^ mice of both sexes (n = 3–4 mice/group) were obtained as described previously [18] from level 3 at the level of the second palatal ridge of the mouse nasal cavity [19]. Tissue sections were rehydrated with 0.1M phosphate buffered saline (PBS), permeabilized with 0.01–0.3% triton x-100 and blocked with 5% BSA or 10% normal donkey serum. Tissue sections were incubated with goat anti-olfactory marker protein (OMP, 1∶1000, Waco Chemicals, Richmond, VA), mouse anti-growth-associated protein 43 (GAP43, 1∶100, Sigma-Aldrich, St. Louis, MO), rabbit anti-cytokeratin 5 (CK5, 1∶100, Abcam, Cambridge, MA), mouse anti-MASH1 (1∶30, BD Pharmingen, San Diego, CA), or rabbit anti-proliferation cell nuclear antigen (PCNA, 1∶50, Abcam, Cambridge, MA), overnight at 4°C. Immunoreactivity was detected by FITC- or TRITC-conjugated donkey anti-goat, mouse or rabbit immunoglobin (1∶50 or 1∶200, Jackson ImmunoResearch Lab, West Grove, PA). For detection of PCNA and MASH1, antigen retrieval was performed before permeabilization by heating sections in a citrate buffer (pH = 6) in a microwave oven (700 W; 2×6 min low power). Detection of BrdU was as described previously using rat anti-BrdU (1∶100, Abcam, Cambridge, MA) [20]. NPY immunoreactivity was assessed as described previously using a tyramide signaling amplification kit (Invitrogen/Molecular Probes, Eugene, OR) and rabbit anti-NPY antibody (1∶50, Bachem, Torrance, CA) [20]. The nuclei were counterstained with Vectashield mounting medium for fluorescence with DAPI (Vector Laboratory, Burlingame, CA). Immunoreactivity or GFP fluorescence was visualized on an Olympus FV1000 confocal laser scanning microscope (Pleasant Valley, PA). Antibody specificity was examined by omitting the primary antibody or secondary antibody. No immunoreactivity was observed in any of the controls.

The number of GAP43^+^, Cy5^+^, MASH1^+^, PCNA^+^, BrdU^+^ cells in the ecto-turbinate 2 and endo-turbinate II on three coronal sections of OE between levels 3 and 4 in each animal were counted by an experimenter blinded to the treatments and genotypes. Data was normalized to the length of OE on which the immunoreactive cells were scored and expressed as number per linear millimeter OE. We used a stereological approach to estimate the quantity of OMP^+^ neurons given their large numbers. The percent volume density of OMP^+^ cells was calculated in one coronal section of OE between levels 3 and 4 in each animal using STEPanizer software (www.stepanizer.com, [21]). At 6 regions in the ecto-turbinate, 4 locations in the endo-turbinate II and 1 location in the septum, a small 130×130 µm 144-point overlay was randomly placed (total area analyzed = 16900 µm^2^/location). The volume density of OMP^+^ cells was determined by manual point counting and expressed as the percentage of the ratio of the number of test points hitting OMP-immunoreactive OSNs, divided by the total number of points hitting the olfactory epithelium.

### Olfactory Epithelial Explant Culture

Glass coverslips (Fisher Scientific, Pittsburgh, PA) placed in a 24 well plate were coated with 50 µg/ml fibronectin (Sigma Aldrich, St. Louis, MO) made in Neurobasal-A media (Invitrogen, Carlsbad, CA) and incubated at 37°C and 5% CO_2_ for 12–18 hr. OE tissue pooled from 6 neonatal (postnatal day 3–5) C57BL/6 mice and OE tissue collected separately from 6 individual neonatal transgenic (IP3R3^+/−^ and IP3R3^−/−^) mice was placed in 4°C sterile-filtered Hanks’ Balanced Salt Solution containing 50 µg/ml gentamycin and 6 mg/ml glucose (Invitrogen, Carlsbad, CA), and washed twice with 4°C sterile-filtered Neurobasal-A “growth media” containing 20 µg/ml B27 supplement and 50 µg/ml gentamycin (Invitrogen, Carlsbad, CA), and 0.5 mM L-glutamine and 13.0 mM NaCl (Sigma Aldrich, St. Louis, MO). Tissue was sliced into ca. 200×200 µm explants and single explants were placed on fibronectin-coated coverslips and incubated at 37°C and 5% CO_2_ without media for 30 min to allow for adherence. Warm (37°C) growth media supplemented with 5 ng/ml FGF2 (Invitrogen, Carlsbad, CA) was added to each well (500 µl total volume), and growth media was changed every 2 days. BrdU (50 µg/ml, Sigma Aldrich, St Louis, MO) was added to the growth media at day 4 and 6. At day 8, cultures were rinsed with warm (37°C) 0.1 M PBS, fixed with sterile-filtered 4% paraformaldehyde for 10 min at room temperature and stored in 0.1M PBS at 4°C until used for immunocytochemistry.

OE explants were removed from the coverslip and the surrounding cells growing outside the OE explant were incubated with 2 M HCl for 1 hr at 37°C to denature DNA, followed by a rinse with 0.1M sodium tetraborate +0.1 M boric acid (pH 8.5). Cells were permeabilized with 0.05% Tween-20 (Sigma Aldrich, St. Louis, MO) in 0.1M PBS and incubated in blocking solution (2% normal donkey serum and 0.05% Tween-20) followed by rat anti-BrdU (1∶200 in blocking solution) for 2 hr at room temperature. Cells were washed and then incubated with donkey anti-rat immunoglobulin conjugated to TRITC (1∶500, Jackson ImmunoResearch Lab) for 2 hrs. For co-localization of BrdU with GAP43 or OMP, cells were incubated with a mixture of rat anti-BrdU and mouse anti-GAP43 or goat anti-OMP antibodies followed by donkey anti-rat immunoglobulin conjugated to TRITC and anti-mouse or goat immunoglobulin conjugated to FITC (1∶500, Jackson ImmunoResearch Lab) for 2 hrs. The coverslips were mounted on Superfrost Plus slides with Vectashield Mounting Medium (Vector Labs, Burlingame, CA). FITC and TRITC were excited at 465–495 and 530–560 nm, respectively, and emissions were collected at 515–555 and 573–648 nm, respectively using a Nikon TE2000-U inverted fluorescence microscope (Nikon, Melville, NY). No immunoreactivity was observed when the primary antibody or secondary antibody was omitted. Cells that grew outward and were dispersed from the explant were quantified [22]. The number of immunoreactive (IR) cells and the total number of cells (counted in bright field) were tabulated and averaged from five non-overlapping fields per coverslip acquired using a 20×objective (0.5 n.a.) and Metamorph 7.5 (Molecular Devices, Sunnyvale, CA). Data are expressed as the percentage (mean+SEM) of IR cells to total cells. Each group included 3–6 coverslips.

### Bulbectomy Procedure

Unilateral olfactory bulb ablation was performed on adult IP3R3^+/−^ and IP3R3^−/−^ mice as described previously [23]. Following surgery, the animals underwent daily intranasal aspiration of saline vehicle or ATP (400 nmol/kg) for 3 or 7 days. BrdU was administered (i.p., 144 mg/kg total) at 6 and 3 hours prior to OE tissue collection at 24 hours after the last intranasal aspiration.

### Statistical Analysis

Student’s t-test, and one-way ANOVA followed by the Newman-Keuls post-hoc test was performed using Prism 5 (Graphpad Software, San Diego, CA). Two-way ANOVA or repeated measures two-way ANOVA was performed using GB-Stat v9.0 (Dynamic Microsystems, Inc., Silver Spring, MD). Following two-way ANOVA, the Newman-Keuls post hoc test was used when the number of groups was ≤3 and the Tukey Kramer post-hoc test was used when the number of groups was >3 to avoid exceeding the acceptable limit of type I error.

## Results

We previously demonstrated that ATP, released following injury [24], stimulates the release of NPY [7] that subsequently induces proliferation of progenitor cells in mouse OE [20]. Here, we investigate (1) the purinergic receptor subtypes that mediate NPY release, (2) the role of the IP3R3 receptor in mediating ATP-induced NPY release, and subsequently (3) the role of the IP3R3-mediated NPY release in maintaining normal tissue homeostasis and in initiating regeneration following injury.

### Activation of P2X_7_ and P2Y_2_ Purinergic Receptors Induces NPY Release in the OE

**Figure 1:**
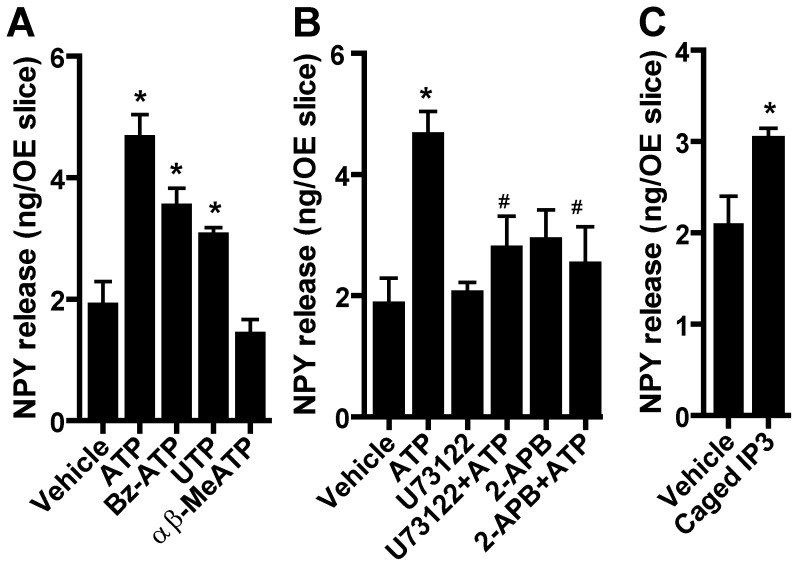
The release of neurotrophic factor NPY following injury simulation is mediated by a purinergic receptor, phospholipase C, and IP3/IP3 receptor pathway. (A) P2X_1,7_ and P2Y_2_ receptors mediate NPY release. Neonatal OE slices from Swiss Webster mice were incubated with vehicle (0.2% DMSO), non-selective P2 purinergic receptor agonist (ATP, 50 µM), P2X_1,7_ agonist (BzATP, 50 µM), P2Y_2,4,6_ agonist (UTP, 50 µM) or P2X_1,2/3,3_ agonist (αβ-MeATP, 50 µM) for 1 hour and the levels of NPY in media were measured by ELISA. *, p<0.05 vs. vehicle (one-way ANOVA followed by Newman-Keuls post-hoc test; n = 6, 4, 4, 4 and 3 replications, respectively.) (B) ATP-induced NPY release is PLC- and IP3 receptor-dependent. Neonatal OE slices from Swiss Webster mice were pre-incubated with vehicle (0.2% DMSO), PLC inhibitor (U73122, 100 µM), or IP3 receptor inhibitor (2-APB, 100 µM) for 45 min prior to addition of vehicle (0.2% DMSO) or ATP (50 µM). Media was collected after 1 hr and the levels of NPY in media were measured by ELISA. *p<0.01 vs. vehicle; #p<0.05 vs. ATP (two-way ANOVA followed by Newman-Keuls post-hoc test; n = 6, 4, 4, 4, 5 and 5 replications, respectively.) (C) IP3 induces NPY release. Neonatal OE slices from Swiss Webster mice were incubated with vehicle (0.09% pluronic acid +0.45% DMSO) or caged iso-IP3/PM (caged IP3, 1.5 µM) and slices were illuminated with unfiltered light from a xenon arc lamp (30 min) to uncage IP3. Media was collected 1 hr later and the levels of NPY were measured by ELISA. p = 0.02 (Student’s t-test; n = 3 and 4 replications.).

To investigate the purinergic receptor-mediated mechanisms underlying NPY release in the OE, we used exogenous purinergic analogs. Incubation of OE slices obtained from outbred Swiss Webster mouse neonates with the non-specific P2X and P2Y agonist ATP and the P2Y_2,4,6_ agonist UTP (50 µM) for 1 hour significantly increased NPY release compared to vehicle (p<0.05, Fig. 1A), corroborating our previously reported results [7]. Moreover, incubation of OE slices with P2X_1,7_ agonist Bz-ATP (50 µM) significantly increased NPY release (Fig. 1A; p<0.05). However, P2X_1,2/3,3_ agonist αβ-MeATP (50 µM) did not induce NPY release from OE slices (Fig. 1A; p>0.05 v. vehicle). These data indicate that Bz-ATP-stimulated NPY release is via activation of P2X_7_ receptors. To verify that ATP induces NPY release in Swiss Webster adult mouse OE, whole turbinates were incubated in vehicle or ATP (50 µM) for 1 hr. ATP significantly increased the amount of NPY released compared to control (72.1±8.7 v. 46.8±6.1 pg/mg OE; n = 8 turbinates from 8 mice, p = 0.04, Student’s t-test). These data indicate that ATP stimulates the release of NPY in neonatal and adult OE. Given the reported expression of P2X_1,3,4,5,7_ and P2Y_1,2_ purinergic receptors in the mouse OE [15], [25], [26], these data indicate that activation of P2X_7_ and P2Y_2_ but not P2X_1,2/3,3_ purinergic receptors stimulates NPY release in the OE.

### IP3R3 has a Role in Injury-simulated NPY Release in the OE

NPY is predominantly expressed in IP3R3 containing microvillous cells in the OE [5], [7]. Thus, we investigated whether IP3 receptors were involved in ATP-induced NPY release. Incubation of OE slices obtained from Swiss Webster neonates with the non-specific IP3 receptor inhibitor 2-APB (100 µM) did not alter the basal control level of NPY release (p>0.05, Fig. 1B). However, 2-APB significantly blocked ATP-induced increase of NPY release compared to ATP alone (p<0.05, Fig. 1B). Incubation of OE slices with the phospholipase C (PLC) inhibitor U73122 (100 µM; 15 min) to inhibit the formation of IP3 by cleavage of phosphatidylinositol 4,5 bisphosphate did not alter NPY release compared to control, but U73122 significantly blocked the ATP-induced increase of NPY release (p<0.05, Fig. 1B). Finally, we directly stimulated OE slices with IP3 via incubation with caged iso-IP3/PM (1.5 µM). Uncaging IP3 by UV illumination (30 min) significantly increased the levels of NPY in the culture media compared to vehicle incubation (p<0.02, Fig. 1C). Collectively, these data indicate that injury-simulated NPY release in the OE is PLC- and IP3 receptor-dependent.

**Figure 2:**
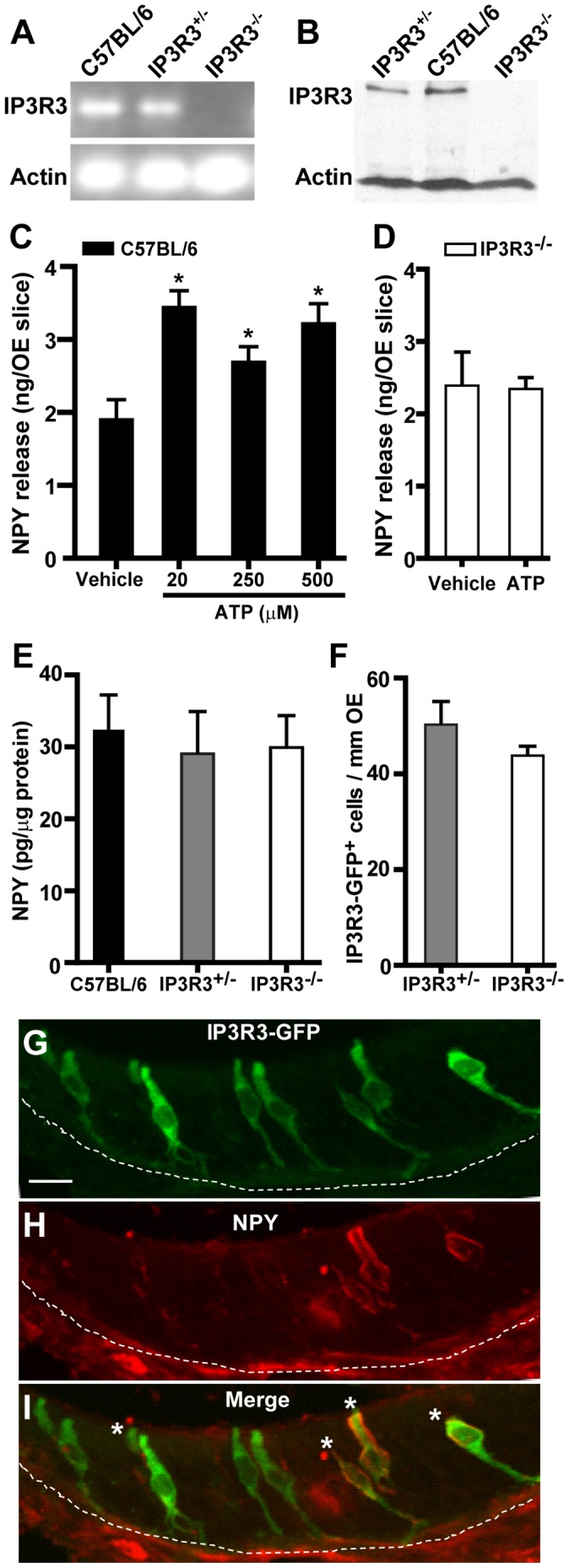
The release of neurotrophic factor NPY following injury simulation is impaired in IP3R3^−/−^ mice. (**A–B**) The OE of IP3R3^−/−^ mice does not express IP3R3. IP3R3 mRNA (A) and protein (B) in the OE of adult C57BL/6, IP3R3^+/−^ and IP3R3^−/−^ mice were measured by PCR and Western blot analysis. (**C**) ATP induces NPY release in the OE of C57BL/6 mice. Neonatal OE slices from C57BL/6 mice were incubated with vehicle (0.2% DMSO) or ATP (20, 250 or 500 µM) for 1 hour. * P<0.01 or 0.05 vs. vehicle (one-way ANOVA followed by Newman-Keuls post-hoc test; n = 4, 4, 4 and 5 replications, respectively.) (**D**) ATP does not induce NPY release in the OE of IP3R3^−/−^ mice. Neonatal OE slices from IP3R3^−/−^ mice were incubated with vehicle (0.2% DMSO) or ATP (50 µM) for 1 hour (p = 0.9, Student’s t-test; n = 5 replications, each.) (**E**) Protein levels of NPY in the OE of C57BL/6, IP3R3^+/−^ and IP3R3^−/−^ mice as quantified by ELISA (p = 0.6, one way ANOVA with Neuman-Keuls post-hoc test; n = 6, 4, 6 mice, respectively.) (**F**) The number of IP3R3-tauGFP^+^ cells in the OE of IP3R3^+/−^ and IP3R3^−/−^ mice are comparable (p = 0.09 Student’s t-test, n = 17 and 22 sections from 6 and 7 mice, respectively.) (**G–I**) GFP^+^ cells (F) and NPY^+^ cells (G) co-localize (H; *) in the OE of IP3R3-tau GFP mice. Scale bar = 10 µm.

Next, we focused on whether the IP3R3 subtype specifically mediates injury-simulated NPY release using IP3R3^+/−^ and IP3R3^−/−^ mice. Measurement of the IP3R3 gene (Fig. 2A) and protein (Fig. 2B) confirmed that IP3R3^−/−^ mice lack IP3R3 expression in the OE. In order to confirm that there are no strain differences in ATP-induced NPY release in the OE, we incubated neonatal OE slices from C57BL/6 (i.e., same genetic background as IP3R3-tauGFP) mice with vehicle or ATP (20, 250 or 500 µM). ATP (20–500 µM) significantly induced NPY release compared to vehicle (p<0.05, Fig. 2C), confirming that C57BL/6 mice can also be used to measure ATP-stimulated NPY release. Incubation of OE slices from IP3R3^−/−^ mice with ATP (50 µM) did not significantly alter the NPY release compared to saline vehicle (p = 0.9, Fig. 2D), indicating that ATP-induced NPY release is impaired in the OE of IP3R3^−/−^ mice. Note that the unstimulated physiological levels of NPY released from OE slices from Swiss Webster, C57BL/6, and IP3R3^−/−^ mice were comparable (Fig. 1A–C v. 2C v. 2D). Taken together, these data suggest that IP3R3 is involved in ATP-induced NPY release.

The loss of ATP-induced NPY release in IP3R3^−/−^ mice could be due to a reduction in either the number of IP3R3-expressing cells or in NPY levels. To test this, we quantified the number of IP3R3-tauGFP^+^ cells and protein levels of NPY in the OE of C57BL/6, IP3R3^+/−^ and IP3R3^−/−^ mice under basal (unstimulated) conditions. The protein levels of NPY and the number of IP3R3-tauGFP^+^ cells were comparable between all mice (Fig. 2E–F, p>0.05). In IP3R3^+/−^ mice, 31.3±4.9% (mean±SEM) of IP3R3-tauGFP^+^ cells co-localize with NPY^+^ cells (Fig. 2G–I), consistent with the number of NPY^+^ cells in unstimulated Swiss Webster mice [20]. Collectively, these data indicate that the reduction in NPY release observed in the IP3R3^−/−^ mice was not due to a decrease in NPY expression or number of IP3R3-containing cells.

### IP3R3^−/−^ Mice have a Significant Reduction in Progenitor Cells and Immature Neurons

**Figure 3:**
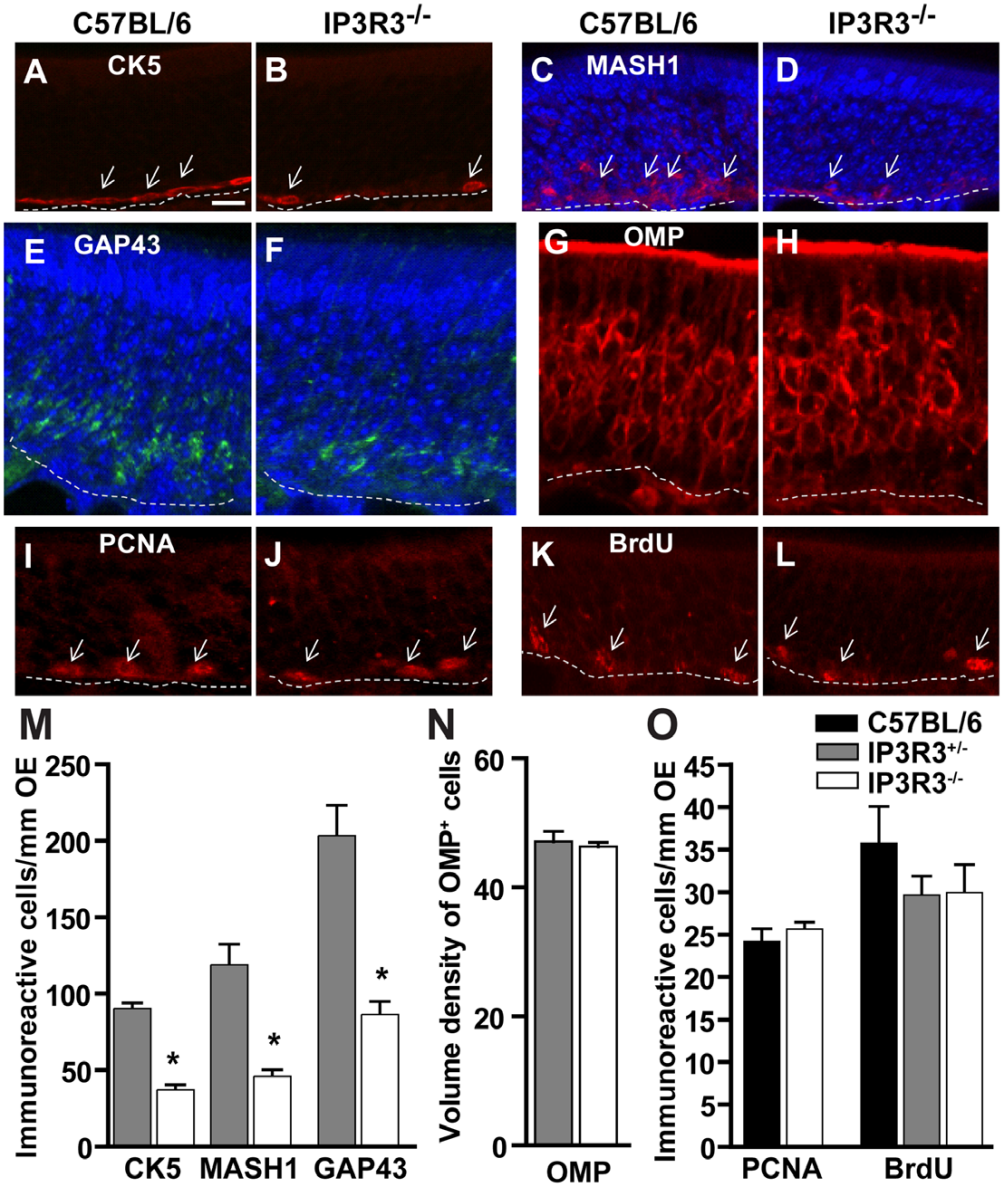
IP3R3^−/−^ mice have fewer basal cells but a normal rate of proliferation in the OE. (**A–L**) Representative immunoreactivity to cellular markers in adult C57BL/6 (A, C, E, G, I, K) and IP3R3^−/−^ (B, D, F, H, J, L) mice: (A–B) horizontal basal cell marker cytokeratin 5 (CK5), (C–D) proneural transcription factor MASH1, (E–F) immature neuronal marker GAP43, (G–H) mature neuronal marker OMP, (I–L) proliferation cell marker PCNA and BrdU. DAPI (blue) demarcates the nuclei. Scale bar, 10 µm, shown in A is relevant for A–I. (**M**) The number of CK5^+^ HBCs, MASH1^+^ progenitor cells and GAP43^+^ immature neurons in the OE of IP3R3^−/−^ mice are significantly reduced. *, p<0.01 vs. respective control in C57BL/6 (Student’s t-test for each cell marker; n = 9–12 sections from 3–4 mice. Refer to legend in (O) for (M–O). (**N**) The number of OMP^+^ neurons in the OE of IP3R3^−/−^ is similar to C57BL/6 mice (p = 0.39, Student’s t-test; n = 9–12 sections from 3–4 mice.) (**O**) The rate of proliferation measured by PCNA expression and BrdU incorporation is comparable in the OE of IP3R3^−/−^, IP3R3^+/−^ and C57BL/6 mice. p>0.05, Student’s t-test and one way ANOVA, respectively; n = 9–12 sections from 3–4 mice. Legend refers to M–O.

A change in the amount of released NPY, a neuroproliferative factor, could alter the microenvironment and ultimately affect the behavior and fate of the basal progenitor cells. Thus, we first examined the complement of cells in the OE of adult C57BL/6 and IP3R3^−/−^ mice. Compared to C57BL/6 mice, the number of cells expressing cytokeratin 5 (CK5), found in horizontal basal cells, MASH1, a proneural transcription factor found in a subpopulation of global basal cells and GAP43, a marker of immature olfactory sensory neurons, was significantly reduced in the OE of IP3R3^−/−^ mice (p<0.01, Fig. 3A–F, M). This indicates that in the IP3R3^−/−^ mice, there are fewer progenitor cells and subsequently fewer cells that have started the neuronal differentiation process. However, the number of OMP^+^ mature olfactory sensory neurons in IP3R3^−/−^ mice was not significantly changed (p = 0.39, Fig. 3G–H, N). This suggests that the pool of progenitor cells in the IP3R3^−/−^ mice are sufficient to maintain the number of mature neurons. We next examined the rate of proliferation and show that the number of proliferation cell nuclear antigen (PCNA)-immunoreactive (IR) cells and BrdU-IR proliferating cells in the basal layer in the OE of IP3R3^−/−^ mice was not changed when compared to the control C57BL/6 and IP3R3^+/−^ mice (p>0.05, Fig. 3I–L, O). Collectively, these data indicate that although the number of progenitor cells in the OE of IP3R3^−/−^ mice is reduced, there is an adequate population of basal cells involved in cell proliferation that maintains the stable number of mature olfactory sensory neurons.

**Figure 4:**
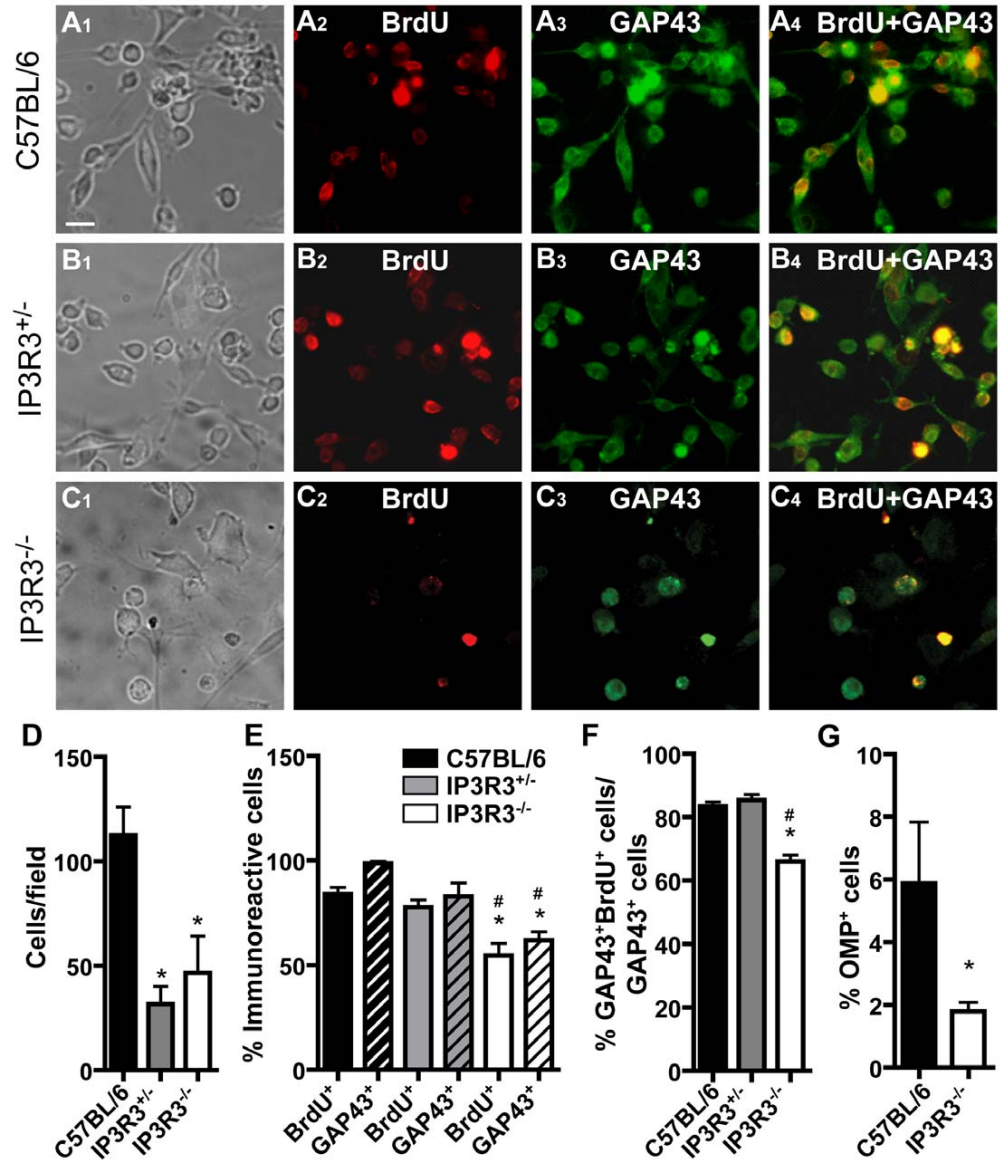
The capacity of basal cells to differentiate is compromised in the OE of IP3R3^−/−^ mice. (**A–C**) Representative images of OE explant cultures from neonatal (A) C57BL/6, (B) IP3R3^+/−^ and (C) IP3R3^−/−^ mice exposed to BrdU (50 µg/ml) from day 4–8. Cells surrounding the OE explant immunoreactive to antibodies directed against BrdU (A2–C2), immature neuron marker GAP43 (A3–C3), to both BrdU and GAP43 (A4–C4), or mature neuron marker OMP (data not shown) were quantified. Scale bar = 50 µm. (**D**) There was a significantly lower number of cells surrounding the explants from IP3R3^+/−^ and IP3R3^−/−^ mice than compared to C57BL/6 mice. *, p<0.05 v. C57BL/6 (One way ANOVA with Newman-Keuls post-hoc test; n = 3, 3 and 5 coverslips, respectively.) Refer to legend in E. (**E**) BrdU^+^ and GAP43^+^ cells in the explant culture from IP3R3^−/−^ mice were significantly lower compared to C57BL/6 and IP3R3^+/−^ mice. *, p<0.01 v. C57BL/6; #, p<0.05 v. IP3R3^+/−^ (Two way ANOVA with Newman-Keuls post-hoc test; n = 3, 4, 3 coverslips, respectively). Legend refers to D, F–G. (**F**) Co-localization of BrdU^+^ and GAP43^+^ cells in the explant culture from IP3R3^−/−^ mice was significantly lower compared to IP3R3^+/−^ and C57BL/6 mice. *, p<0.01 v.C57BL/6, #, p<0.001 v. IP3R3^+/−^ (One way ANOVA with Newman-Keuls post-hoc test; n = 3 coverslips, each.) (**G**) OMP^+^ cells in the explant culture from IP3R3^−/−^ mice were significantly lower compared to that of C57BL/6 mice. *, p<0.02 (Student’s t-test; n = 3 and 6 coverslips, respectively).

To further determine the capacity of basal cells to proliferate and differentiate, we used olfactory epithelial explant cultures isolated from C57BL/6, IP3R3^+/−^ and IP3R3^−/−^ mice. The progenitor cells in an OE tissue explant can proliferate, migrate away from the explant and differentiate into cells that exhibit characteristics similar to neurons, basal cells and progenitor cells in vivo [27]. The total cell number in the cultures from C57BL/6 mice was significantly higher than that from IP3R3^+/−^ and IP3R3^−/−^ mice (Fig. 4A1–C1, D; p<0.05), suggesting that there are fewer progenitor cells in the IP3R3^+/−^and IP3R3^−/−^ OE. GFP^+^ microvillous cells were not observed migrating from the explants from IP3R3^+/−^ and IP3R3^−/−^ mice (data not shown). We measured BrdU incorporation as a marker for proliferation from OE explants cultured in BrdU-supplemented growth media (days 4–8). Cell proliferation in IP3R3^−/−^ cultures was significantly reduced compared to C57BL/6 and IP3R3^+/−^ cultures (Fig. 4A2–C2, E; p<0.01 and p<0.05, respectively). The ability of progenitor cells to differentiate into neurons was quantified by tabulating the number of immature neuron marker GAP43-immunoreactive cells, and of mature neuronal marker OMP-immunoreactive cells. GAP43^+^ cells in the cultures from IP3R3^−/−^ mice were significantly lower than that of C57BL/6 and IP3R3^+/−^ cultures (Fig. 4A3–C3, E; p<0.01 v. C57BL/6 and p<0.05, v. IP3R3^+/−^). In order to determine whether GAP43^+^ immature neurons were “born” in vitro, we quantified the number of cells in which nuclei were labeled with BrdU and cytoplasm was labeled with GAP43 and normalized the data to the total number of GAP43^+^ cells. A significant reduction in GAP43^+^/BrdU^+^ cells was observed in IP3R3^−/−^ cultures compared to C57BL/6 and IP3R3^+/−^cultures (Fig. 4A4–C4, F; p<0.01 v. C57BL/6, p<0.001 v. IP3R3^+/−^). Although the number of OMP^+^ cells in C57BL/6 and IP3R3^−/−^ mice was relatively low at day 8 in vitro, the number of OMP^+^ cells in IP3R3^−/−^ mice was significantly lower than that in C57BL/6 mice (Fig. 4G; p<0.02). Taken together, these data indicate that the capacity of basal cells to proliferate and differentiate is compromised in IP3R3^−/−^ cultures.

### The Response to Injury is Compromised in the OE of IP3R3^−/−^ Mice

**Figure 5:**
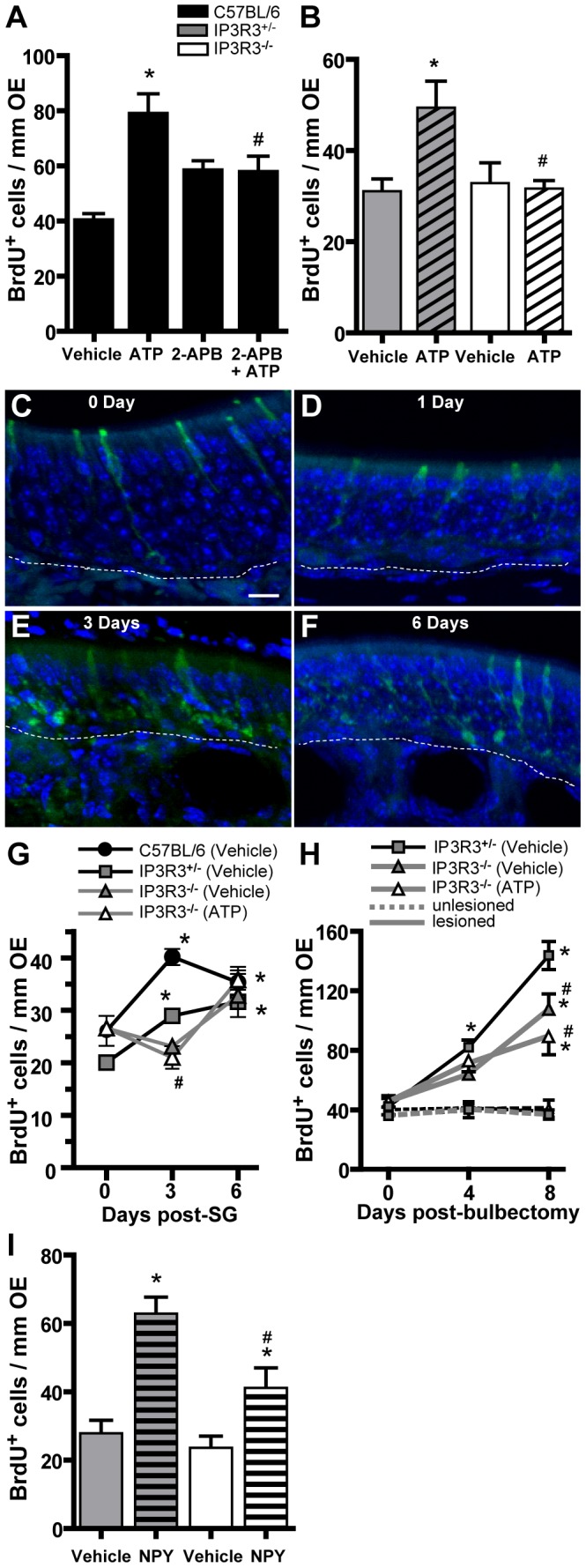
The response to injury is compromised in the OE of IP3R3^−/−^ mice. (**A–E**) BrdU incorporation was quantified following treatments described below (mean +/− SD reported). (**A**) IP3 receptors mediate ATP-induced increase in cell proliferation. C57BL/6 mice intranasally aspired vehicle (saline) or the IP3 receptor inhibitor 2-APB (400 nmol/kg) 30 min prior to vehicle (saline) or ATP (400 nmol/kg). Tissue was collected 48 hours post-instillation of ATP. *, p<0.01 vs. vehicle, ^#^p<0.05 vs. ATP (two-way ANOVA followed by Newman-Keuls post-hoc test; n = 9–12 sections from 3–4 mice.) Legend is for A, B, G–I. (**B**) ATP-induced increase in cell proliferation is abolished in the OE of IP3R3^−/−^ mice. IP3R3^+/−^ and IP3R3^−/−^ mice intranasally aspired vehicle (saline) or ATP (400 nmol/kg) and tissue was collected 48 hours post-instillation of ATP. *, p<0.05 vs. vehicle in IP3R3^+/−^, #, p<0.05 vs. ATP in IP3R3^+/−^ (two-way ANOVA followed by Newman-Keuls post-hoc test; n = 9–18 sections from 3–6 mice.) Refer to legend in (A). (**C–F**) IP3R3^+/−^ cells do not degenerate following satratoxin G exposure. Representative confocal z-stack images of the OE with IP3R3-tauGFP expressing microvillous cells (green) and nuclei labeled with DAPI (blue) at 0, 1, 3 and 6 days following satratoxin G instillation (100 µg/kg) are shown. Scale bar, 20 µm. (**G**) C57BL/6, IP3R3^+/−^ and IP3R3^−/−^ mice intranasally aspired vehicle (saline) or satratoxin G (100 µg/kg) followed by daily aspiration of saline vehicle or ATP (400 nmol/kg) and tissue was collected 3 and 6 days post-administration of satratoxin G. Satratoxin G-induced increase in cell proliferation in the OE of IP3R3^−/−^ mice is compromised and exogenous ATP does not increase proliferation. *, p<0.05 vs. day 0 of the respective group; #, p<0.05 vs. vehicle in C57BL/6 (two-way ANOVA followed by Tukey Kramer post-hoc test; n = 7–12 sections from 3–4 mice.) (**H**) Bulbectomy-induced increase in cell proliferation in the OE of IP3R3^−/−^ mice is compromised and exogenous ATP does not increase proliferation. Unilateral bulbectomy was performed in IP3R3^+/−^ and IP3R3^−/−^ mice and tissue collected 4 and 8 days post-surgery. Tissue was collected at day 0 from sham-treated mice. Mice intranasally aspired vehicle (saline) or ATP (400 nmol/kg) for 3 or 7 days following surgery. Solid lines indicate lesioned side and dashed lines indicate unlesioned side. BrdU incorporation in the lesioned side was significantly higher than in the unlesioned side at 4 and 8 days in all groups (p<0.01, not indicated in figure). *, p<0.01 vs. day 0 of respective group; #, p<0.01 vs. lesion side of IP3R3^+/−^ (two-way ANOVA followed by Tukey Kramer post-hoc test; n = 9–12 sections from 3–4 mice.) (**I**) Exogenous NPY significantly increases cell proliferation in the OE of IP3R3^−/−^ mice. IP3R3^+/−^ and IP3R3^−/−^ mice intranasally aspired vehicle (saline) or NPY (4 nmol/kg) and tissue was collected 48 hours post-instillation of NPY. *, p<0.05 vs. respective vehicle; #, p<0.01 vs. NPY in IP3R3^+/−^ mice (two-way ANOVA followed by Newman-Keuls post-hoc test; n = 9–12 sections from 3–4 mice.) Refer to legend in A.

Next, we examined the response of IP3R3^−/−^ mice to injury, which requires a significant population of basal cells to proliferate and differentiate to replace dying olfactory sensory neurons. Injury was first simulated by using ATP. C57BL/6, IP3R3^+/−^ and IP3R3^−/−^ mice intranasally aspired saline vehicle or ATP (400 nmol/kg) and BrdU-incorporation was quantified in the OE 48 hours post-administration. ATP significantly increased the number of BrdU^+^ cells compared to vehicle control in the OE of C57BL/6 mice (p<0.01, Fig. 5A). Pre-treatment of C57BL/6 mice with IP3 receptor inhibitor 2-APB (400 nmol/kg) did not alter the number of BrdU^+^ cells in the OE of saline vehicle-instilled control animals but significantly blocked the ATP-induced increase in BrdU incorporation (p<0.05, Fig. 5A), indicating that IP3 receptors are involved in ATP-induced increase of cell proliferation in the OE. ATP also significantly increased the number of BrdU^+^ cells compared to vehicle control in the OE of heterozygous IP3R3^+/−^ mice (p<0.05, Fig. 5B). Importantly, ATP had no effect on the number of BrdU^+^ cells in the OE of IP3R3^−/−^ mice compared to saline vehicle. The number of BrdU^+^ cells in the OE of IP3R3^−/−^ mice following ATP treatment was significantly lower than that of heterozygous IP3R3^+/−^ mice (p<0.05, Fig. 5B), indicating ATP-mediated cell proliferation is impaired in IP3R3^−/−^ mice.

We next used satratoxin G, a black mold toxicant that induces apoptosis in olfactory sensory neurons with consequent ATP release [28], as an upstream method to induce injury. IP3R3^+/−^ and IP3R3^−/−^ mice intranasally aspired saline vehicle or satratoxin G (100 µg/kg) and BrdU-incorporation was quantified in the OE at 3 and 6 days post-instillation. The presence of IP3R3-tau GFP^+^ cells was observed in IP3R3^+/−^ mice at 1, 3, and 6 days post-satratoxin G exposure (Fig. 5C–F), indicating that satratoxin G does not eliminate IP3R3-expressing microvillous cells, and validating the use of this injury model for this experiment. In C57BL/6 and IP3R3^+/−^ mice, satratoxin G significantly increased the number of BrdU^+^ cells in the OE at both 3 and 6 days post-instillation (p<0.05, Fig. 5G). In contrast, in the OE of IP3R3^−/−^ mice satratoxin G treatment slightly decreased BrdU incorporation at 3 days compared to 0 days (p>0.05, Fig. 5G), but at 6 days BrdU incorporation was increased such that there was no significant difference in BrdU^+^ cells between IP3R3^+/−^ and IP3R3^−/−^ mice. These data indicate there is a delay in the response to injury in IP3R3^−/−^ mice in the initial early proliferative wave, possibly due to the reduction in basal cell numbers, but that the proliferative response is able to “catch up” to the IP3R3^+/−^ response by 6 days. Intranasal aspiration of ATP (400 nmol/kg) following satratoxin G did not significantly alter the number of BrdU^+^ cells in the OE of IP3R3^−/−^ mice compared to vehicle treated IP3R3^−/−^ mice (Fig. 5G), indicating that ATP activation is upstream of the IP3R3-mediated release of NPY. Overall, these data indicate that the ability to increase cell proliferation following satratoxin G-induced injury is altered in the OE of IP3R3^−/−^ mice.

To further confirm the role of the IP3R3 in the injury response, we performed unilateral bulbectomy on IP3R3^+/−^ and IP3R3^−/−^ mice and examined the levels of BrdU^+^ cells in the OE at 4 and 8 days post-surgery. Bulbectomy induces synchronous apoptosis of olfactory sensory neurons followed by increased cell proliferation in the OE with no consequential ATP release [23], [29]. Bulbectomy significantly increased BrdU-incorporation in the OE on the lesioned side compared to the unlesioned side in IP3R3^+/−^ and IP3R3^−/−^ mice at both 4 and 8 days post-surgery (p<0.01, Fig. 5H). In IP3R3^+/−^ mice, bulbectomy significantly increased the number of BrdU^+^ cells in the OE at both 4 and 8 days compared to 0 days (p<0.01, Fig. 5H). In IP3R3^−/−^ mice, bulbectomy significantly elevated BrdU-incorporation in the OE only at 8 days compared to 0 days (p<0.01, Fig. 5D), corroborating the delay in satratoxin G-induced proliferation in IP3R3^−/−^ mice (Fig. 5C). Furthermore, the number of BrdU^+^ cells in the OE of IP3R3^−/−^ mice was significantly lower than that of IP3R3^+/−^ mice at 8 days post-surgery (p<0.05). In addition, in IP3R3^−/−^ mice, intranasal aspiration of ATP following bulbectomy did not significantly alter the bulbectomy-induced increase in BrdU^+^ cells compared to vehicle treatment (p>0.05, Fig. 5H). These data indicate that the bulbectomy-induced increase in cell proliferation in the OE of IP3R3^−/−^ mice is impaired and additional stimulation of proliferation using ATP has no effect, unlike that which was observed previously in wild-type mice [28]. Taken together, data from all three injury models (ATP simulation, satratoxin G and bulbectomy) indicate that the injury-induced increase in cell proliferation in the OE of IP3R3^−/−^ mice is impaired. Overall, the IP3R3 is involved in the injury-induced increase of cell proliferation in the OE.

### NPY Induces Proliferation in IP3R3^−/−^ Mice

Our data showing that IP3R3 mediates ATP-induced NPY release and a subsequent increase of cell proliferation in the OE suggests that IP3R3-mediated NPY release may mediate ATP- or injury-induced increases in cell proliferation. Therefore, we intranasally administered NPY (4 nmol/kg) to mice to mimic ATP-induced NPY release and measured subsequent cell proliferation. In the OE of IP3R3^+/−^ and IP3R3^−/−^ mice, NPY significantly increased the number of BrdU^+^ cells (p<0.05, Fig. 5I). Cell proliferation was significantly lower in the NPY-treated IP3R3^−/−^ mice compared to the NPY-treated IP3R3^+/−^ mice (p<0.01, Fig. 5I). These data support that IP3R3-mediated NPY release is involved in injury-stimulated cell proliferation in the OE.

## Discussion

The mechanisms that regulate progenitor cell self-renewal and maintain the population of neural progenitor cells throughout adulthood are not fully understood. We do know that neurotrophic factors produced by both neural and non-neural cells regulate neural progenitor cell proliferation and differentiation. However, the exact mechanisms underlying neurotrophic factor secretion are not known. We have shown that activation of IP3R3 mediates the release of the neurotrophic factor NPY in mouse OE. Furthermore, in the absence of IP3R3, NPY release is diminished, and the number of CK5^+^ basal cells, MASH1^+^ progenitor cells, and immature neurons is reduced. Under physiological conditions, this reduction in immature neurons and progenitor cells does not affect the rate of proliferation; however, following injury or damage, the regenerative, proliferative response is reduced. Addition of exogenous neuroproliferative neurotrophic factor NPY to IP3R3^−/−^ mice can potentiate the ability to proliferate. Thus, IP3R3^+^ microvillous cells, via NPY release, have an important role in the maintenance of the neural stem cell population under normal conditions and following injury.

### Role of IP3R3 and Purinergic Receptors in Secretion of NPY

We previously demonstrated that ATP and UTP stimulate NPY release from mouse neonatal OE slices via activation of purinergic receptors [7]. Our current data indicate that activation of P2X_7_ and P2Y_2_ receptors induces NPY release. P2X_7_ receptors have low sensitivity to the concentrations of ATP used in these experiments (50 µM; EC_50_>100 µM) [29], indicating that activation of P2X_7_ receptors by ATP did not contribute significantly to the observed NPY release. However, extracellular ATP concentrations in the millimolar range can occur under pathological conditions, suggesting that the P2X_7_ receptor may have a role in NPY release following injury. Metabotropic P2Y_2_ receptors couple to the phospholipase C (PLC)/IP3 intracellular pathway and have a high sensitivity to ATP (EC_50_ = 240 nM [30]), suggesting that P2Y_2_ receptors mediate NPY release under both physiological and pathophysiological conditions.

Previous studies identified links between IP3R2 and IP3R3 and saliva secretion from salivary glands, digestive enzyme secretion in the pancreas [12] and olfactory mucus secretion from nasal glands [17]. Here, we show that evoked NPY release is completely absent in the OE of IP3R3^−/−^ mice, indicating that IP3R3 is the only mediator involved in ATP-induced NPY release. NPY is released presumably from secretory granules located in IP3R3^+^ microvillous cells, the predominant cell type that expresses high levels of NPY in the adult OE [5], [7]. IP3 induces increases in intracellular calcium in the OE (Hegg, unpublished observations) that may have a role in NPY release. For instance, IP3 receptors are present in secretory granules [16] and IP3-mediated release of calcium from granules can facilitate calcium-dependent mobilization, docking and fusion of secretory granules. Conversely, the calcium stores in rat insulinoma secretory granules are IP3 insensitive, indicating that not all secretory granules have IP3 receptors or display characteristics of regulated calcium stores [31]. It is unclear if calcium has a role in NPY release. There is no difference in the response rate, peak amplitude, and characteristics of ATP-induced intracellular calcium signaling in IP3R3-tauGFP expressing cells from IP3R3^+/−^ and IP3R3^−/−^ OE tissue ([15] and unpublished data). These data suggest that ATP-induced IP3R3-dependent NPY release in the OE is calcium-independent. In addition, although activation of ionotropic P2X_3_ receptors expressed in IP3R3^+/−^ microvillous cells increases intracellular calcium through calcium influx [15], the P2X_3_ receptor agonist αβ-MeATP failed to induce NPY release, supporting a calcium-independent ATP-induced NPY release mechanism. The mechanism underlying calcium-independent IP3R3-mediated NPY release is unknown. Calcium-independent neurotransmitter release has been observed previously, [32]–[34] e.g., by direct activation of the secretory apparatus by the polyvalent cation ruthenium red [35]. Furthermore, cAMP promotes calcium-independent insulin release by a direct interaction with the secretory machinery [36]. However, the use of BzATP, a potent ionotropic P2X_7_ agonist, induces increases in intracellular calcium [37] and NPY release, supporting a calcium-dependent mechanism of NPY release. P2X_7_ receptor-mediated NPY release could be similar to P2X_7_ receptor-evoked calcium-dependent exocytotic release of ATP observed in the OE and neuroblastoma cells [37], [38]. Alternatively, activation of P2X_7_ receptors could directly elicit NPY release in a calcium-independent manner. For instance, the N-terminal of the P2X_7_ receptor is linked to activation of extracellular signal-regulated kinases (ERK1/2) independent of calcium influx [39]. In the OE, ATP-mediated NPY release is linked to activation of the ERK1/2 pathway [9]. Further investigation is required to fully elucidate the role of calcium in IP3R3-dependent NPY release.

### Role for IP3R3^+^ Microvillous Cells in Tissue Homeostasis

The neural progenitor cell microenvironment that regulates progenitor cell self-renewal and maintains the population of neural progenitor cells throughout adulthood is not fully understood. NPY stimulates cell proliferation in the OE [6]. NPY-expressing IP3R3^+^ microvillous cells have cytoplasmic processes that extend towards the NPY Y1 receptor-expressing stem cells [5]–[7], [20]. Thus, the neuroproliferative effect of NPY in the OE may be mediated by paracrine secretion of NPY. A significant reduction in olfactory neural precursor proliferation occurs in NPY-deficient and NPY Y1-deficient mice [6], [8]. Although the population of immature neurons was not assessed, the number of mature neurons was significantly reduced in NPY-deficient and NPY Y1-deficient mice, indicating neurogenesis is compromised in the absence of NPY and NPY Y1 receptors [6], [8]. Notably, there are conflicting results in NPY-deficient and NPY Y1-deficient mice. In NPY-deficient mice, there is a significant increase in MASH1^+^ globose basal cells, however, not all of the MASH1^+^ cells obtained from NPY-deficient mice generate neurospheres, indicating that not all of the MASH1^+^ cells observed in the OE of NPY-deficient mice are globose basal cells [8]. In NPY Y1-deficient mice there are fewer MASH1^+^ globose basal cells [8]. In NPY-deficient mice, the location of keratin^+^ horizontal basal cells is disrupted, with some cells appearing below the basement membrane. Keratin^+^ horizontal basal cells appear morphologically normal in NPY Y1-deficient mice [8], indicating that NPY Y1 receptors are either not present in horizontal basal cells or not involved in maintaining the horizontal basal cell population. These results, in general, corroborate our observations of fewer keratin^+^ horizontal basal cells and MASH1^+^ globose basal cells in IP3R3^−/−^ mice. Previous reports did not observe morphological changes in the OE of IP3R3-deficient mice, although quantification was not performed [17]. Clearly, IP3R3-mediated NPY release has a role in neural stem cell homeostasis.

The number of basal cells in IP3R3^−/−^ mice was decreased despite the fact that the proliferation rate and the number of mature OMP^+^ neurons were unchanged. This phenomenon could be due to a reduction in the survival of basal cells and immature olfactory sensory neurons or the upregulation of either NPY Y1 receptors on basal cells or positive regulators of neurogenesis to compensate for the decrease in basal cells. Possibilities of positive regulators include fibroblast growth factor and transforming growth factor [40], amidated peptides [41], leukemia inhibitory factor [42] and OMP [43]. The overall implications of these results are that an appropriate population of neural stem cells is maintained by IP3R3^+^ microvillous cells by the production and release of trophic factor NPY.

### Role for IP3R3^+^ Microvillous Cells in Regeneration

IP3R3^+^ microvillous cells were speculated to function in the regeneration of the OE based on the sole expression of neuroproliferative agent NPY in the adult OE [4], [5]. Here, we now provide direct evidence indicating that IP3R3^+^ microvillous cells, via IP3R3-dependent NPY release, promote adult neurogenesis following two forms of injury. Satratoxin G-induced injury causes ATP release and the promotion of NPY-mediated cell proliferation [7], [18], [20], [28]. Olfactory bulb ablation stimulates globose basal cell proliferation [44]–[46] in a manner that does not involve the release of ATP [23]. Both types of injury caused a delay in regenerative proliferation in IP3R3^−/−^ mice, although bulbectomy does not induce ATP-mediated NPY release. Thus, this suggests that the delay in proliferation can be accounted for by the reduction in progenitor cells in IP3R3^−/−^ mice. However, our results showing that exogenous NPY can potentiate proliferation in IP3R3^−/−^ mice lends support to the idea that IP3R3-mediated NPY release is also a key component required for neuroregeneration following injury.

### Conclusions

We have identified key signaling components (ATP, NPY, and IP3R3) that regulate both tissue homeostasis and injury-induced neuroregeneration. Furthermore, these results establish a function for microvillous cells: to maintain the population of neural stem cells and to promote adult neurogenesis via IP3R3-dependent NPY release. Without the contributions of microvillous cell-mediated NPY release, homeostatic neurogenesis and neuroregeneration following injury are impaired. These results identify microvillous cells and IP3R3 as new, innovative pharmacological targets to manipulate adult OE neuroregeneration.
